# Submacular Hemorrhage Following Aflibercept Intravitreal Injection: A Report of Two Cases

**DOI:** 10.7759/cureus.27255

**Published:** 2022-07-25

**Authors:** Chloe Khoo, Erin Flynn, Preet Sohal, Rheem Al Shabeeb, Baha El Khatib, Marena Patronas

**Affiliations:** 1 Ophthalmology, George Washington University School of Medicine and Health Sciences, Washington D.C., USA; 2 Internal Medicine, George Washington University School of Medicine and Health Sciences, Washington D.C., USA

**Keywords:** complications, submacular hemorrhage, intravitreal injection, aflibercept, age-related macular degeneration

## Abstract

Anti-vascular endothelial growth factor (anti-VEGF) injections are the most effective treatment for exudative age-related macular degeneration (AMD). However, both bevacizumab and ranibizumab have been reported to cause submacular hemorrhage (SMH) in the treatment of exudative AMD. Aflibercept has also been reported to cause SMH but only in the treatment of polypoidal choroidal vasculopathy and not exudative AMD.

This case series presents two patients with exudative AMD who developed SMH after treatment with aflibercept injections. The first patient is an 84-year-old female with exudative AMD in both eyes who presented with SMH four days after an aflibercept injection in her right eye. The second patient is a 77-year-old female who presented with exudative AMD in her left eye and SMH one month following an aflibercept injection. This case series shows that SMH in patients treated for exudative AMD is a rare yet possible complication of aflibercept injection that requires further research to establish its incidence and risk factors.

## Introduction

Age-related macular degeneration (AMD) is the leading cause of blindness in adults over the age of 65. Dry or non-exudative AMD is the more prevalent subtype described by a small buildup of extracellular material called “drusen” in Bruch's membrane. Wet or exudative AMD is a more debilitating condition described by abnormal blood vessels and leakage within weeks to months of diagnosis [1]. Anti-vascular endothelial growth factor (anti-VEGF) injections used to treat exudative AMD include aflibercept and ranibizumab, which are Food and Drug Administration (FDA) approved while bevacizumab is used off-label. Anti-VEGF injections are generally safe procedures with a reported endophthalmitis prevalence of 0.2% per injection [2-3].

Submacular hemorrhages (SMH) are a collection of blood between the retinal pigment epithelium (RPE) and the sensory retina derived from either the choroidal or retinal blood circulation. It represents a serious complication that can happen in the natural course of exudative AMD secondary to a number of etiologies, including rupture of choroidal blood vessels or disruption of the retinal pigmented epithelium [4]. Visual outcomes after SMH are generally poor due to irreversible photoreceptor damage, where the initial and final visual acuity is thought to correlate with the size and thickness of the hemorrhage. Goverdhan et al. reported a case series of 54 patients where visual acuity in 80% of the eyes worsened from 20/240 to 20/1, 250 at the 24-month follow-up [5].

For patients with a history of continuous, scheduled anti-VEGF injections for exudative AMD treatment, SMH has previously been reported with the use of bevacizumab and ranibizumab but not aflibercept [6-9]. SMH following aflibercept injection has been reported in cases of polypoidal choroidal vasculopathy (PCV) [9]. To our knowledge, this is the first study that describes two cases of SMH following monthly aflibercept injection in patients with exudative AMD.

## Case presentation

Case 1

**Figure 1:**
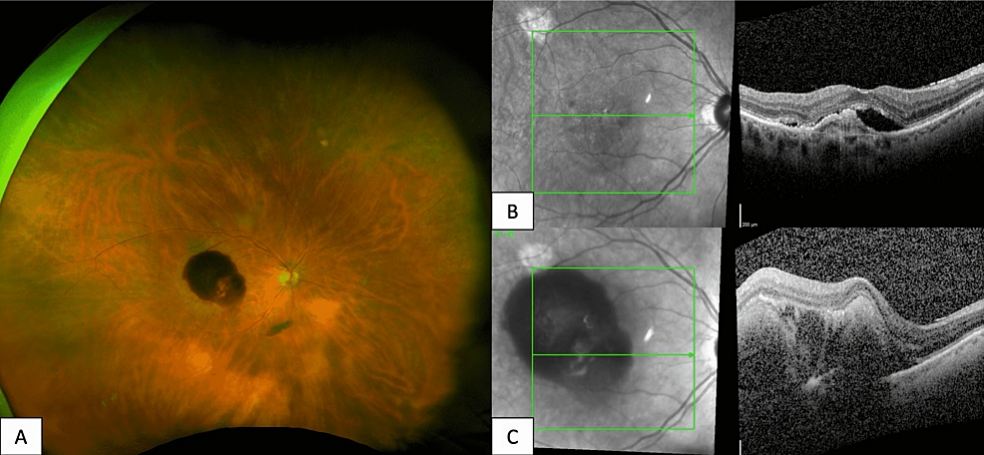
Fundus Photography and Optical Coherence Tomography of Subretinal Hemorrhage Following Aflibercept Injection An 84-year-old female with exudative AMD in the right eye. Optos fundus photography of the right eye (A) reveals a healthy optic nerve with diffuse subretinal macular hemorrhage, mild attenuation of vessels, and a 1DD choroidal nevus along the superotemporal arcade. Optical coherence tomography (OCT) macula of the right eye prior to aflibercept injection (B) reveals subretinal fluid with an adjacent pigment epithelial detachment. OCT macula of the right eye taken four days after aflibercept injection (C) reveals sub-macular elevation with subretinal and sub-retinal pigment epithelium (RPE) thickening.

An 84-year-old female, with a history of AMD in both eyes, presented with exudative AMD in her right eye and non-exudative AMD in her left eye in 2010. Initially, her right eye was treated with monthly intravitreal aflibercept. Before starting aflibercept 15 months before, this patient had received several doses of ranibizumab and bevacizumab in her right eye; however, aflibercept was found to be more efficacious in her case. In 2017, the non-exudative AMD in her left eye converted to exudative AMD, and she subsequently started receiving monthly aflibercept injections as well. In 2019, the right eye developed a large SMH four days after receiving an aflibercept injection. She complained of decreased vision in her right eye. On exam, her visual acuity had decreased from 20/25 to 20/200. Dilated fundus examination of the right eye revealed a large diffuse subretinal macular hemorrhage (Figure 1).

Optical coherence tomography (OCT) showed a large sub-macular elevation with both subretinal and sub-RPE thickening (Figure 1). She was subsequently switched from aflibercept to bevacizumab injections every four to eight weeks. Given her age, she declined further surgical intervention. At the six-month follow-up, her visual acuity was 20/400, and the submacular hemorrhage had dehemoglobinized and diminished in size.

Case 2

A 77-year-old female with a history of non-exudative AMD in her right eye and exudative AMD in her left eye presented one month after aflibercept injection with blurry vision in her left eye. The patient had been on aflibercept for 19 months. On exam, her visual acuity had declined from 20/70 to count fingers from six inches. Dilated fundus exam revealed a large subretinal macular hemorrhage. OCT showed increased subretinal thickening and sub-RPE elevation with a pigment epithelial detachment (PED). She was subsequently switched to bevacizumab and underwent a 25-gauge pars plana vitrectomy with a subretinal injection of tPA. Her submacular hemorrhage resolved and on the last follow-up assessment, visual acuity improved to 20/200. She underwent one session of photodynamic therapy (PDT) due to persistent intraretinal fluid that resolved following PDT and is currently receiving monthly bevacizumab injections. She also underwent one session of photodynamic therapy (PDT) due to persistent intraretinal fluid that resolved following PDT.

## Discussion

Aflibercept has a higher binding affinity to VEGF than other anti-VEGF analogs like bevacizumab or ranibizumab, and its efficacy in managing exudative AMD has been well-demonstrated [10-11]. A study of 25 eyes with SMH secondary to exudative AMD showed that intravitreal injection of aflibercept is an effective treatment where it improved best corrected visual acuity (BCVA), decreased central foveal thickness, decreased the area of SMH, and stabilized vision in 96% of eyes [12]. Studies have also reported its effectiveness in treatment-resistant exudative AMD and in reducing PEDs [13-14].

Given the well-documented effectiveness of aflibercept, this case series presents a rare and unexpected finding following the use of this medication. In two of our patients, aflibercept was discontinued and both patients were switched to bevacizumab. Additionally, both patients underwent different treatment modalities for their SMH, with only our second case who received surgical management with hemorrhage resolution but still poor visual acuity at 20/200.

However, it is important to note that the progression of exudative AMD is associated with choroidal neovascularization (CNV), which can lead to SMH. Therefore, determining the significance of the development of SMH in our patients treated with aflibercept as occurring secondary to the medication or due to progression of their AMD is difficult.

To date, there are studies showing that CNV treated with intravitreal bevacizumab and ranibizumab is associated with the development of acute SMH [5-6]. In one study, eyes with occult CNV > or = to 15 mm as measured on fluorescein angiography (FA) treated with intravitreal bevacizumab were more likely to develop SMH compared with intravitreal ranibizumab [6]. However, there are very few reports documenting SMH after intravitreal aflibercept.

Another possibility for SMH after intravitreal injection would be an RPE tear. RPE tears have previously been identified as a side effect of intravitreal anti-VEGF injections [7] and can subsequently lead to SMH. These have been reported to occur not just with aflibercept use but also with bevacizumab and ranibizumab [8-13].

However, we hypothesize that tachyphylaxis towards aflibercept was the cause of the SMH because of the long duration of treatment with aflibercept in both cases. While tachyphylaxis is a phenomenon that is well-documented in bevacizumab and ranibizumab with studies estimating an annual occurrence rate of 10% [14] and 2-7.7% [14-15], respectively, very little has been published on tachyphylaxis associated with aflibercept.

A 2019 retrospective cohort study utilized aflibercept as a first-line anti-VEGF intravitreal injection in 313 treatment-naive eyes with a mixture of the pathology of exudative AMD (with classic and occult type CNV), polypoidal choroidal vasculopathy, and retinal angiomatous proliferation. The study further found that 8.9% of study participants met the criteria for tachyphylaxis to aflibercept at an annual rate of 2.2-4.0% [16]. All cases of tachyphylaxis developed in cases with CNV beneath the RPE. These cases were AMD with occult CNV and PCV and had lower rates of retinal edema. Researchers hypothesized that perhaps the RPE created a barrier that was more impenetrable to anti-VEGF injections in occult CNV, and perhaps the presence of intraretinal edema indicates the disruption of RPE allowing for the better penetration of anti-VEGF injections [16]. Interestingly, both of our patients had occult type CNV with fibrovascular PED and minimal to no intraretinal edema, which is consistent with reports of tachyphylaxis.

The existing literature on tachyphylaxis to aflibercept suggests that an increased number of injections and duration of treatment were associated also with a greater rate of tachyphylaxis. This is consistent with research on tachyphylaxis in both bevacizumab and ranibizumab. Both patients in this study had a long duration of treatment with aflibercept - the first having been on aflibercept for nine years and the second, over two years. However, more research is required to identify the association of SMH with aflibercept use in treating exudative AMD and the predisposing risk factors to developing SMH.

## Conclusions

Submacular hemorrhage after intravitreal aflibercept for exudative AMD is a rare complication. It appears that, like tachyphylaxis to ranibizumab and bevacizumab, an increased amount of injections, as well as the increased duration of treatment, predisposes patients to this risk. Furthermore, patients with exudative AMD characterized by occult CNV, CNV beneath the RPE, and fewer instances of retinal edema were more at risk for tachyphylaxis with aflibercept.
